# Successful airway management with combined use of a McGRATH^TM^ MAC videolaryngoscope and fiberoptic bronchoscope in a patient with congenital tracheal stenosis diagnosed in adulthood

**DOI:** 10.1186/s40981-021-00452-w

**Published:** 2021-06-09

**Authors:** Shoko Hasegawa, Kenichiro Koda, Masashi Uzawa, Haruka Kimura, Rie Kimura, Takayuki Kitamura

**Affiliations:** Department of Anesthesiology, Toho University Sakura Medical Center, 564-1 Shimoshizu, Sakura, Chiba, 285-8741 Japan

**Keywords:** Difficult airway management, Tracheal intubation, Videolaryngoscope, Fiberoptic bronchoscope, Complete tracheal rings, Pulmonary artery sling

## Abstract

**Background:**

Most patients with congenital tracheal stenosis (CTS) develop respiratory symptoms early in life. CTS remaining undiagnosed until adulthood is rare.

**Case presentation:**

A 51-year-old female was scheduled for cardiovascular surgery. She had undergone laparoscopic surgery 3 years earlier and was found to have a difficult airway. Postoperatively, she was diagnosed with CTS. For the current cardiovascular surgery, combined use of a McGRATH^TM^ MAC videolaryngoscope and fiberoptic bronchoscope allowed sufficient visualization of the glottis and trachea, resulting in successful intubation.

**Conclusions:**

CTS patients have a high probability of difficult intubation. Our experience suggests the efficacy of combined use of a videolaryngoscope and fiberoptic bronchoscope for airway management in CTS patients.

## Background

Congenital tracheal stenosis (CTS) is a rare, life-threatening disorder [1, 2]. Due to lack of the membranous trachea and trachealis muscle, tracheal cartilage forms complete tracheal rings, resulting in fixed tracheal narrowing [1, 2]. Most CTS patients develop respiratory symptoms early in life [1]. CTS that remains undiagnosed until adulthood is extremely rare. Here, we report successful airway management with combined use of a McGRATH^TM^ MAC videolaryngoscope (Aircraft Medical Ltd., Edinburgh, UK) and fiberoptic bronchoscope (FOB) in an adult CTS patient.

## Case presentation

A 51-year-old female (158.5 cm, 51.4 kg) was scheduled for surgical repair of pulmonary artery sling and partial anomalous pulmonary venous return (PAPVR). She had previously undergone atrial septal defect (ASD) closure at 6 years old. The surgical record indicated that her trachea was smaller than age appropriate, and there had been difficulty in tracheal intubation. Since the age of 33 years, she was treated as bronchial asthma.

She underwent laparoscopic bilateral salpingo-oophorectomy at 48 years old. The flow-volume curve in a pulmonary function test was consistent with fixed-type upper airway obstruction, and peak expiratory flow was decreased to 40% of the predicted value, while forced expiratory volume % in 1 s (%FEV_1.0_) was within normal limits. Tracheal narrowing was overlooked, although it was evident on retrospective inspection of chest X-ray. Anesthesia was induced with propofol (100 mg), fentanyl (100 μg), remifentanil (0.1 μg/kg/min), and rocuronium (40 mg). The glottis was visualized by direct laryngoscopy using a size-3 Macintosh blade and was classified as grade 2b according to the modified Cormack-Lehane classification; however, it was impossible to advance a standard polyvinylchloride-cuffed tracheal tube (Shiley^TM^ endotracheal tube with a TaperGuard^TM^ cuff; Covidien Japan, Tokyo, Japan) with a 7.0-mm internal diameter (ID) and 9.5-mm outer diameter (OD). A second attempt using a cuffed tube with a 6.5-mm ID and 8.9-mm OD also failed. The trachea was finally intubated with a cuffed tube with a 6.0-mm ID and 8.2-mm OD. The tube was inserted to 20 cm from the incisors. The lungs were well ventilated without inflation of the cuff. All attempts were performed by a Japanese Society of Anesthesiologists (JSA) board certified anesthesiologist. The surgery was completed uneventfully, and her trachea was extubated immediately after surgery.

**Fig. 1:**
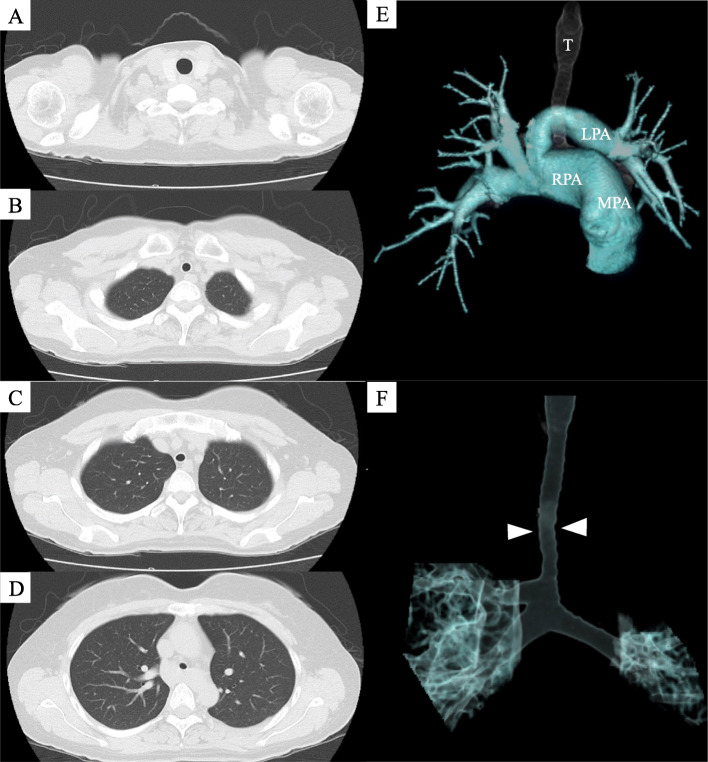
Preoperative imaging findings. Chest computed tomography showed stenosis over the entire length of the trachea with complete tracheal rings (**A**-**D**). Reconstructed three-dimensional images showed a pulmonary artery sling (**E**) and tracheal bronchus (**F**). The narrowest part of the trachea (arrowheads) was 6 mm in diameter (**F**). The left pulmonary artery originated from the right pulmonary artery and encircled the trachea (**E**). MPA, main pulmonary artery; RPA, right pulmonary artery; LPA, left pulmonary artery; T, trachea

**Fig. 2:**
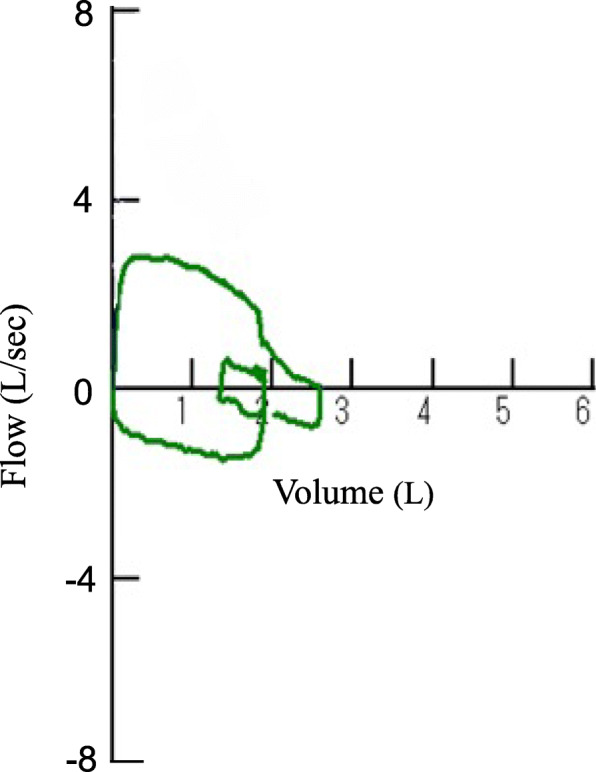
Preoperative pulmonary function test. The flow-volume curve showed a characteristic fixed upper airway obstruction pattern

Postoperatively, chest computed tomography revealed stenosis over the entire length of the trachea with complete tracheal rings (Fig. 1A-D), PAPVR, and pulmonary artery sling (Fig. 1E), and she was diagnosed with CTS. The internal transverse diameter of the narrowest part of trachea was 6 mm. The right upper pulmonary vein drained into the superior vena cava. A tracheal bronchus was also identified (Fig. 1F). Transesophageal echocardiography showed residual ASD. Pulmonary to systemic blood flow ratio was 1.54. Further dilatation of the left pulmonary artery was expected to exacerbate the effects of CTS. Therefore, surgical repair of the cardiovascular malformations was scheduled. A fixed-type upper airway obstruction with decreased peak expiratory flow to 36% of the predicted value was reconfirmed (Fig. 2).

We considered that intubation closer to the carina carried the risk of increase in airway resistance as well as tracheal injury, and shallow intubation should be applied. Inflation of the cuff was required to avoid accidental extubation, while also taking care to prevent compression of the vocal cords by the cuff. We planned to secure an approximately 1-cm margin between the cuff and the vocal cords. We chose a cuffed tube with a 6.0-mm ID and 8.2-mm OD: the length from the tip of tube to the proximal edge of cuff was 6 cm. The length from the vocal cords to the carina was 15 cm. The internal transverse diameter of trachea 7 cm from the vocal cords was 10 mm. We planned to advance the tip of tube to 7 cm from the vocal cords. Anesthesia was induced with midazolam (5 mg), fentanyl (200 μg), remifentanil (0.2 μg/kg/min), and rocuronium (50 mg). One anesthesiologist (a senior doctor in training) performed laryngoscopy using a McGRATH^TM^ MAC videolaryngoscope. The videolaryngoscope provided full view of the glottis. The tube was advanced until the cuff just passed through the glottis. The anesthesiologist continued observation of the glottis using the videolaryngoscope, while another anesthesiologist (a fellow of JSA) inserted a FOB via the tube. The tube was advanced under bronchoscopic guidance. This enabled insertion of the tube to 19 cm from the incisors. With the aid of the FOB, we confirmed that there was no tracheal bleeding, and the tip of tube did not contact the trachea. The cuff was inflated with air so that internal pressure was 15 cmH_2_O. Under videolaryngoscopic visualization, we withdraw the tip of FOB from the inflated cuff to the vocal cords and confirmed an approximately 1-cm margin. General anesthesia was maintained using sevoflurane, fentanyl, remifentanil, and rocuronium. We commenced continuous administration of dopamine (1-3 μg/kg/min) and dobutamine (2-6 μg/kg/min) at the time of the weaning from cardiopulmonary bypass and continued it until the end of surgery. The surgery was completed uneventfully. The tracheal tube was removed on the second postoperative day (POD-2). She did not complain of respiratory distress. She was transferred to the general ward on POD-3, and was discharged on POD-22.

## Discussion

More than 75% of CTS patients have other anomalies [1]. Pulmonary artery sling is the most common abnormality [1, 2], in which the left pulmonary artery originates from the right pulmonary artery and encircles the right main bronchus and distal trachea [2]. This patient was found to have difficult airway during laparoscopic gynecological surgery. Subsequently, she was diagnosed as CTS with cardiovascular malformations, and surgical repair was scheduled. Safe and atraumatic airway management is a must for surgery under cardiopulmonary bypass. We succeeded in tracheal intubation by combined use of a videolaryngoscope and FOB in this patient. The videolaryngoscope provided a clear glottic view. The FOB was useful for confirming the atraumatic intubation. Additionally, we could validate the margin between the cuff and the vocal cords.

CTS remaining undiagnosed until adulthood is extremely rare. A literature search identified 16 CTS patients diagnosed at the age of 20 years or older [3–18]. Table 1 shows the summary of the 17 patients, including the present case.

**Table 1:** Reported cases of congenital tracheal stenosis diagnosed in adulthood

Case	Age/gender	Height (cm)/weight (kg)	Respiratory symptoms	Initial detection of CTS	Minimum diameter in trachea (mm)	Reference
1	60/F	151.2/39.6	Dyspnea	Bronchoscopy	5	[3]
2	57/M	154/68	Impaired exercise tolerance	Chest X-ray, chest CT	NR	[4]
3	45/F	160/59	None	Difficult intubation	NR	[5]
4	25/F	Small stature/45	None	Difficult intubation	6	[6]
5	39/F	162/65	None	Difficult intubation	10	[7]
6	42/F	151/52	None	Difficult intubation	6	[8]
7	21/F	NR/NR	Acute respiratory failure, wheezing	Difficult intubation	7	[9]
8	53/F	NR/NR	None	Difficult intubation	5	[10]
9	42/F	150.8/54.8	Dry cough	Chest X-ray, chest CT	6	[11]
10	34/F	NR/NR	Shortness of breath/chest tightness	Chest CT	8	[12]
11	29/F	NR/NR	Dyspnea, wheezing, stridor	Chest CT	7	[13]
12	37/F	NR/morbidly obese	Dyspnea, stridor	Chest CT	6.3	[14]
13	42/F	151.1/38.8	Dyspnea due to pneumothorax	Difficult intubation	NR	[15]
14	23/F	NR/NR	Wheezing, extertional chest pain	Chest X-ray, chest CT	6.8	[16]
15	70/M	NR/NR	None	Difficult intubation	NR	[17]
16	52/F	162/59	None	Difficult intubation	8.27	[18]
17	51/F	158.5/51.4	Wheezing	Difficult intubation	6	Present case

*F* female, *M* male, *CTS* congenital tracheal stenosis, *CT* computed tomography, *NR* not recorded

Seven patients had no respiratory symptoms [5–8, 10, 17, 18]. Among the 10 patients with respiratory symptoms, five patients had asthma-like symptoms [9, 13, 14, 16]. In fact, four patients were treated as bronchial asthma for a long time [9, 14, 16]. Thus, there might be undiagnosed CTS patients among adult patients with prolonged refractory bronchial asthma. Pulmonary function test results were described in six cases [3–5, 11, 14, 18]. The flow-volume curve was consistent with an upper airway obstruction pattern in five patients [3–5, 11, 14], decreased %FEV_1.0_ was reported in two patients [3, 5], and decreased peak expiratory flow was reported in three patients [3, 5, 14]. Ten of the 17 patients underwent surgery [3, 5–8, 10, 15, 17, 18]. The airway was secured by tracheal intubation in seven patients [3, 5–7, 15, 18]. Due to the failure in tracheal intubation, airway was secured using a laryngeal mask airway in two patients [10, 17], and emergency tracheostomy was performed in the remaining patient [8]. In 10 of the 17 patients, difficult intubation was the initial event in the detection of CTS [5–10, 15, 17, 18]. These data suggest that careful inspection of imaging findings and pulmonary function test results are important, and that anesthesiologists should assume the possibility of undiagnosed CTS when facing unexpected difficult intubation. As an alternative method to securing the airway in CTS patients, the choice of a thinner tube and/or applying shallow intubation seemed to be common [5–9, 15, 18].

In this case, combined use of the videolaryngoscope and FOB provided sufficient visualization of the glottis and the trachea, resulting in successful intubation. Sharma et al. reported successful airway management with combined use of a Glidescope® videolaryngoscope and FOB in a patient with Cowden syndrome, one of the characteristic features of which is papillomatous lesions of the airway [19]. The airway was secured without causing bleeding from the papillomatous lesions. We suppose that combined use of a videolaryngoscope and FOB is a practical method for securing the airway in patients with difficult airway.

## Conclusion

We described airway management for an adult CTS patient. CTS patients have a high probability of difficult intubation. Careful assessment and sufficient preparation to secure the airway are important in CTS patients, regardless of whether they have preoperative respiratory symptoms. Our experiences suggest the efficacy of combined use of a videolaryngoscope and FOB for airway management in adult CTS patients.

## Data Availability

Please contact the corresponding author for data requests.

## Abbreviations

CTS: Congenital tracheal stenosis
FOB: Fiberoptic bronchoscope
PAPVR: Partial anomalous pulmonary venous return
ASD: Atrial septal defect
%FEV_1.0_: Forced expiratory volume % in 1 s
ID: Internal diameter
OD: Outer diameter
JSA: Japanese Society of Anesthesiologists
POD: Postoperative day
